# 

**Published:** 1888-06

**Authors:** 


### CONTENTS.

### COMMUNICATIONS.

Dentogony........ 265
Porcelain Crowns........ 285
Deodorants for Iodoform........ 293
Aluminum Cast Dentures........ 294
A Rescued Fragment........ 300
Non-Union of Bone During Pregnancy........ 310
Salmagundi........ 311

### EDITORIAL.

Educational........ 313
American Medical Association........ 314
Book Notice........ 315
Obituary........ 316

